# Habituation to abrupt-onset distractors with different spatial occurrence probability

**DOI:** 10.3758/s13414-022-02531-1

**Published:** 2022-07-18

**Authors:** Matteo Valsecchi, Massimo Turatto

**Affiliations:** 1 Department of Psychology, University of Bologna, Bologna, Italy; 2 Center for Mind/Brain Sciences, University of Trento, Trento, Italy

**Keywords:** Visual search, Attentional capture, Inhibition

## Abstract

Previous studies have shown that abrupt onsets randomly appearing at different locations can be ignored with practice, a result that was interpreted as an instance of habituation. Here we addressed whether habituation of capture can be spatially selective and determined by the rate of onset occurrence at different locations, and whether habituation is achieved via spatial suppression applied at the distractor location. In agreement with the habituation hypothesis, we found that capture attenuation was larger where the onset distractor occurred more frequently, similarly to what has been documented for feature-singleton distractors (the “distractor-location effect”), and that onset interference decreased across trials at both the high- and low-probability distractor locations. By contrast, evidence was inconclusive as to whether distractor filtering was also accompanied by a larger impairment in target processing when it appeared at the more likely distractor location (the “target-location effect”), as instead previously reported for feature-singleton distractors. Finally, here we discuss how and to what extent distractor rejection based on statistical learning and habituation of capture are different, and conclude that the two notions are intimately related, as the Sokolov model of habituation operates by comparing the upcoming sensory input with expectation based on the statistics of previous stimulation.

## Introduction

It has long been known that humans are particularly responsive to sudden luminance changes (i.e., abrupt onsets) occurring in their visual field, which trigger a fast and involuntary orienting of attention (and the eyes) toward the corresponding location (Breitmeyer & Ganz, 1976; Jonides & Yantis, 1988; Sokolov, 1963; Yantis & Jonides, 1984). Abrupt visual onsets, though, are not the only salient elements possessing this power, as color or shape singletons can also attract attention, both when they are not informative of the target location (Theeuwes & Burger, 1998; Turatto & Galfano, 2000, 2001), and when, in the same trial, they never share the same location of the target (Caputo & Guerra, 1998; Theeuwes, 1992). Yet, abrupt onsets appear to be particularly unique in their attentional grabbing power, as they tend to be more potent attractors compared to other feature singletons (e.g., Folk & Remington, 2015; Jonides & Yantis, 1988; Lamy & Egeth, 2003), and because they grab attention even when this is fully allocated in advance at fixation (Pascucci & Turatto, 2015) or at a peripheral location where the target invariably appears (Turatto & Pascucci, 2016).

However, despite abrupt onsets triggering a strong capture of attention, humans can progressively learn to ignore them, thus reducing their interference on target processing (Turatto & Pascucci, 2016; but see Ruthruff et al., 2019). Accordingly, in recent years studies have documented that the unwanted attentional capture triggered by peripheral onset distractors can be largely attenuated with practice, a result that Turatto and his collaborators have claimed to represent an instance of attentional capture habituation (Turatto et al., 2018a, b, 2019; Turatto & Pascucci, 2016). For example, Turatto and Pascucci (2016) have found that (a) the capture reduction across blocks is larger the higher the onset probability (for a similar finding with a color singleton distractor also see Geyer et al., 2008; Müller et al., 2009; Won et al., 2019); (b) when the distractor is re-introduced, after its removal for some trials, the capture response recovers from the habituated level; (c) the onset capture attenuation is context specific (Turatto et al., 2018a, 2019), three findings in agreement with the characteristics of habituation (Dissegna et al., 2021; Rankin, 2000; Thompson, 2009). In addition, Pascucci and Turatto (2015) have shown that within the same trial the amount of capture diminishes as the number of onsets preceding the target increases (but see Ruthruff et al., 2019), which is also in line with the fact that habituation develops as the same stimulation is repeated. As we have argued in previous studies, the reduction of capture observed for repeated visual onset distractors can be straightforwardly explained by habituation mechanisms like those proposed by Sokolov (1960, 1963) or Wagner (1976, 1978, 1979), which, despite some differences, essentially propose that when a stimulus (e.g., the distractor) is repeatedly presented a corresponding neural model is automatically formed in memory, and the more the new stimulation matches the model (i.e. the history of stimulation), the more the response elicited by the stimulus is suppressed and attenuated.

However, other complementary distractor filtering mechanisms, more or less voluntary or experience-based, have been proposed (Chelazzi et al., 2019; Geng et al., 2019; Liesefeld & Müller, 2019). For example, the results from studies using the additional-singleton paradigm (Theeuwes, 1992) and investigating the filtering of a feature-singleton distractor seem to indicate that the attenuation of capture can be achieved by means of suppressive signals applied to the distractor location, probably at the saliency-map level (e.g., Gaspelin et al., 2015; Luck et al., 2021). In particular, the amount of capture elicited by a feature-singleton distractor is inversely related to its rate of occurrence, with less interference observed at the more likely distractor location (the “distractor-location effect”) (e.g., Ferrante et al., 2018; Goschy et al., 2014; Sauter et al., 2018; Wang & Theeuwes, 2018). This result has been attributed to a statistical learning (SL) process by means of which the spatial rate of the distractor occurrence is estimated, and suppression is applied to the salient element location accordingly. In support of the putative suppressive signals exerted at the feature-singleton location is the fact that not only is distraction reduced where the distractor is more likely to appear, but in certain conditions target processing is also more impaired at same location (the “target-location effect”) (Ferrante et al., 2018; Sauter et al., 2018; Turatto & Valsecchi, 2021; Valsecchi & Turatto, 2021; Wang & Theeuwes, 2018; Zhang et al., 2019).

Hence, although previous studies have shown that the capture response elicited by a repetitive abrupt onset appearing equally likely at all display locations can habituate (e.g., Neo & Chua, 2006; Turatto et al., 2018a; Turatto & Pascucci, 2016), it remains to be established whether habituation of onset capture can vary in space, with habituation being affected by the rate of onset occurrence at different locations (i.e., the “distractor-location effect”), and whether this is accompanied by an impairment in the processing of targets appearing at the distractor location (i.e. the “target-location effect”). Three experiments were devised to address these specific questions.

We must clarify from the outset that although SL and habituation are often presented as alternative accounts for distractor rejection (e.g., Chelazzi et al., 2019; Geng et al., 2019), in fact from our standpoint SL is part of the mechanism controlling habituation of the orienting reflex (OR) (Sokolov et al., 2002), and therefore SL and habituation are intimately related notions (see the General discussion section for a more detailed discussion on this point). However, since this issue needs further evidence to be elucidated, we are also open to the possibility that in fact SL may be a different mechanism compared to that controlling habituation (Duncan & Theeuwes, 2020; Ferrante et al., 2018; Geng et al., 2019; Wang & Theeuwes, 2018).

## Experiment 1

To test whether the attentional grabbing power of an onset distractor varies as a function of its rate of occurrence at a given location, we modeled our paradigm after that proposed by Di Caro and Della Libera (2021). Specifically, the experiment consisted of a training phase followed by a test phase, but crucially throughout the experiment the possible locations occupied by the target never coincided with those occupied by the distractor. This paradigm allows us to manipulate the rate of the target and distractor occurrences at a given location in an independent fashion, so that any local imbalance in the distractor spatial probability does not affect the target spatial distribution, as the two events appear at segregated locations. This total independence is pivotal to test suppressive lingering effects on target processing (the “target-location effect”) due only to the spatial statistics of the distractor in the training phase, as done in Experiment 2.

Hence, our display consisted of eight possible locations, each occupied by a placeholder, four indicating the possible target locations (along the vertical and horizontal axis), and four the possible distractor locations (along the oblique axes). The target appeared equally at each of the corresponding locations, whereas during training the distractor was more likely to appear at one location compared to the other three locations (ratio 8:1), an imbalance that should favor the observation of both the “distractor-location effect” and the “target-location effect” (Lin et al., 2020). In the test phase, the distractor was made equiprobable at all locations, so that we could evaluate whether any differential capture between high- and low-probability distractor locations that emerged during training, endured after the distractor probabilities were equalized across locations (Britton & Anderson, 2020; Duncan & Theeuwes, 2020; Sauter et al., 2019).

This lingering “distractor-location effect” has been interpreted as evidence that during training plastic changes occurred in the saliency map because of the differential degree of suppression exerted at the distractor locations (Britton & Anderson, 2020; Ferrante et al., 2018; Goschy et al., 2014; Sauter et al., 2018; Turatto & Valsecchi, 2021; Wang & Theeuwes, 2018), which persisted for some trials after the distractor probability was equalized at all locations.

### Methods

#### Participants

Participants were recruited online through the Prolific service (Prolific Academic Ltd, Oxford, UK). We required participants to be between 18 and 40 years old, to be native English speakers, to have normal or corrected-to-normal vision, and to run the experiment on a desktop computer. We did not record any further information about participants. Three participants were excluded for failing to reach the overall accuracy level of 85% of correct responses, and were replaced by three new participants to achieve the final sample size of 36. We determined our sample size a priori, to match the one in our previous study (Valsecchi & Turatto, 2021), where we also tested SL of distractor location after probability equalization. In that study (Experiment 1) the comparison of RTs between the formerly high- and low-probability locations in the test phase yielded a significant difference with an effect size *d*_*z*_ = 0.528 and estimated power of 0.868, computed using G*Power 3.1.9.7 (Faul et al., 2007).

All participants were informed about the general aim of the experiment, their task, and data handling procedures in the Prolific interface. They gave their consent by agreeing to be directed to the experiment url, and were paid 3.75 GBP for their participation. The experiment lasted approximately 30 min, and all the experiments of the present study were carried out in accordance with the Declaration of Helsinki, and with the approval of the local institutional ethics committee (Comitato Etico per la Sperimentazione con l’Essere Umano, Università degli Studi di Trento, Italy).

#### Stimuli and procedure

The experiment was built using the PsychoPy3 version 2020.1.3 software (Peirce et al., 2019), and run online using the Pavlovia web hosting service (Open Science Tools Limited, Nottingham, UK).

In order to control the retinal size of the stimuli, at the beginning of the experiment we asked participants to position themselves at a distance that was a multiple of the length (84% of the screen width) of a reference segment presented on the screen. Both the stimuli and the reference segment were defined in screen coordinates, so that if a participant performed the experiment on a larger display, this would be compensated by the proportional increase in viewing distance. The size of the stimuli is reported in degrees of visual angle assuming the instructed viewing distance.

**Fig. 1:**
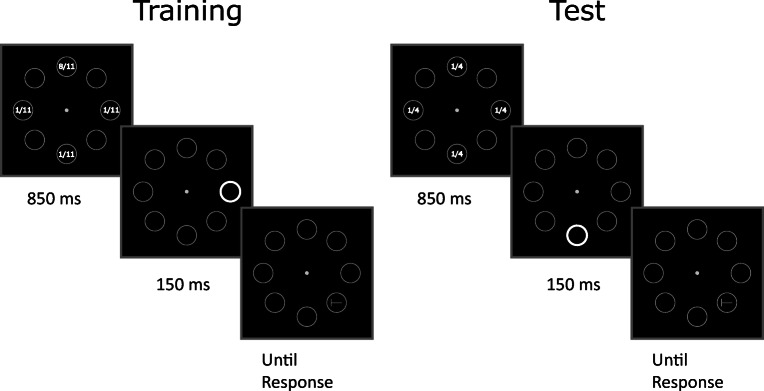
Stimuli and procedure of Experiment 1 depicting a distractor-present trial. Participants reported the orientation of the target letter T (left vs. right) while ignoring the onset distractor appearing on 50% of the total trials. Notice that the locations of the target and distractor were completely segregated within and between trials. The distractor could appear only in the placeholders located along the vertical and horizontal meridians, whereas the target could appear only in the placeholders located along the oblique meridians. During the training phase the distractor was eight times more likely to appear in one of the four possible locations (the numbers inside the placeholders indicate the distractor rate, and were not shown during the experiment). In the example depicted, the higher distractor rate coincided with the upper position along the vertical axis. In the test phase, the distractor probability was equalized at all four locations

The stimuli and procedure are depicted in Fig. 1. The trial began with the onset of a gray central fixation spot (radius .38°), surrounded by eight gray circular placeholders. The placeholders had a radius of 3.4° and were centered at an eccentricity of 7.6° from fixation, and had a line width of 2 pixels. In distractor-present trials, 850 ms after the onset of the initial array one of the placeholders briefly flashed for 150 ms by becoming a white circle (line width 10 pixels). In distractor-absent trials no onset was presented before the occurrence of the target, which consisted of the letter T rotated either clockwise or counterclockwise and appearing inside one of the placeholders (1.5° wide, 2 pixels line width, same gray level as the placeholders) 1,000 ms after the onset of the initial array.

The task required pressing as fast as possible the “G” key if the T was rotated counterclockwise and the “H” key if the T was rotated clockwise, but error minimization was also emphasized. The whole display disappeared after the participant’s response, or 2,500 ms after target onset if no response was provided. In case the response was incorrect or no response was provided within 2,500 ms, the messages “Wrong!” or “Too Slow!” in red letters were presented for 300 ms during the 1-s inter-trial interval.

In order to avoid confounding the SL of distractor and target locations, we presented targets and distractors at non-overlapping locations throughout the experiment. Out of the eight positions defined by the placeholders, four were assigned to the distractor (the positions along the horizontal and vertical meridians), whereas the remaining four positions (corresponding to oblique directions) were occupied by the target.

The experimental design consisted of a practice phase of 28 trials, in which no distractors were presented, followed by a training phase of 352 trials and a test phase of 192 trials. Participants were allowed to rest after performing 176 training trials, and between the training and test phases. In both the training and the test phase, the onset distractor appeared on 50% of the trials. In the training phase the onset distractor was eight times more likely to appear at one location (balanced across participants) compared to each of the remaining three locations. This yielded 176 trials without distractors, 128 trials with the distractor at the high-probability location, and 48 trials at a low-probability location. In the test phase the distractor appeared equally likely at all locations, yielding 96 trials without distractors, 24 trials with distractor in the (formerly) high-probability location, and 72 trials in the (formerly) low-probability locations (24 at each location).

### Results and discussion

Pre-processing of reaction time (RT) data involved removing incorrect responses and applying an outlier-removal procedure based on Median Absolute Deviation (Leys et al., 2013) for each participant and cell of the design, with a threshold of 5 MADs. We further removed RTs faster than 200 ms from the analyses. Overall, we rejected 1.8% of the trials in the training phase, and 1.8% of the trials in the test phase.

#### Training phase

**Fig. 2:**
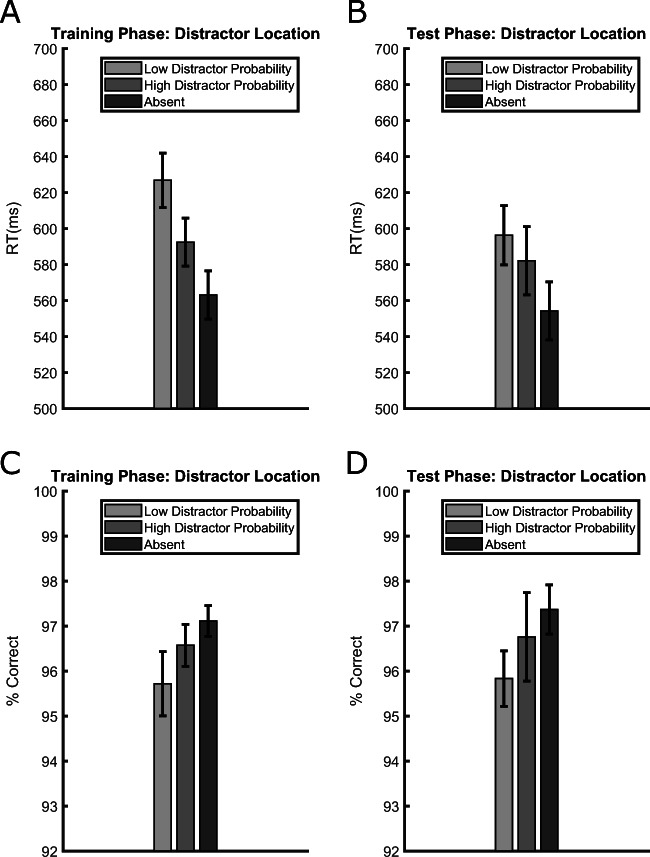
Average reaction times (RTs) (**upper panels**) and accuracies (**lower panels**) in the training (**left panels**) and test (**right panels**) phases of Experiment 1, separately for trials in which the distractor appeared at a low-probability location, at the high-probability location or was absent. Error bars are standard errors of the mean (SEMs)

RT data from the training phase are presented in Fig. 2a, showing that participants were slower in distractor-present trials relative to distractor-absent trials, and this both when the distractor appeared at a low-probability location, *t*(35) = 9.788, *p* < .001, *d*_*z*_ = 1.631, RT difference = 64 ms, and when it appeared at the high-probability location, *t*(35) = 6.314, *p* < .001, *d*_*z*_ = 1.052, RT difference = 29 ms. Crucially, however, the degree of interference was smaller when the distractor appeared at the high-probability location than at a low-probability location, *t*(35) = 8.124, *p* < .001, *d*_*z*_ = 1.354, RT difference = 34 ms, demonstrating that the abrupt onset was ignored better where it appeared more often (the “distractor-location effect”).

Accuracy data from the training phase are presented in Fig. 2c. The results showed an opposite pattern of that emerged for RTs, excluding a possible speed-accuracy trade-off. Only the comparison between the low-probability location and distractor-absent trials was significant, *t*(35) = 2.51, *p* = .016, *d*_*z*_ = 0.418, accuracy difference = 1.39%, whereas the comparison between the high-probability location and the distractor-absent trials, *t*(35) = 1.405, *p* = .168, *d*_*z*_ = 0.234, accuracy difference = 0.54%, and between the high- and low-probability location trials, *t*(35) = 1.324, *p* = .194, *d*_*z*_ = 0.22, accuracy difference = 0.85%, were all not significant.

#### Test phase

An RT pattern similar to the training phase emerged in the test phase (Fig. 2b). Again, compared to the distractor-absent trials, RTs were longer when the distractor was presented either at a previous low-probability location, *t*(35) = 7.318, *p* < .001, *d*_*z*_ = 1.219, RT difference = 42 ms, or at the previous high-probability location, *t*(35) = 4.275, *p* < .001, *d*_*z*_ = 0.712, RT difference = 28 ms. Furthermore, despite the fact that the distractor rate was equalized across locations, a “distractor-location effect” was still evident, as participants were slower when the distractor appeared at a previous low-probability location relative to the previous high-probability location, *t*(35) = 2.287, *p* = .028, *d*_*z*_ = 0.381, RT difference = 14 ms, thus documenting a lingering effect of the local distractor rate from the training phase, similar to what has been shown for feature-singleton distractors (Britton & Anderson, 2020; Ferrante et al., 2018; Goschy et al., 2014; Sauter et al., 2018; Turatto & Valsecchi, 2021; Wang & Theeuwes, 2018).

Accuracy data from the test phase (Fig. 2d) again showed the specular pattern compared to RTs. As in the training phase, only the comparison between the low-probability location and distractor-absent trials was significant, *t*(35) = 3.292, *p* = .002, *d*_*z*_ = 0. 548, accuracy difference = 1.53%, whereas the comparison between high-probability location trials and distractor-absent trials, *t*(35) = 0.645, *p* = .522, *d*_*z*_ = 0.107, accuracy difference = 0.6%, and between high- and low-probability location trials, *t*(35) = 1.043, *p* = .303, *d*_*z*_ = 0.174, accuracy difference = 0.92%, were all not significant.

The present experiment attested that, as in the case of feature-singleton distractors (e.g., Ferrante et al., 2018; Goschy et al., 2014; Sauter et al., 2018; Wang & Theeuwes, 2018), the distracting power of an irrelevant peripheral abrupt onset was attenuated where it appeared more often. In keeping with our previous studies on habituation of onset capture (e.g., Turatto & Pascucci, 2016), we interpret this finding as showing that the habituation mechanism leading to capture attenuation can be spatially selective, and capable of promoting two concurrent different levels of habituation determined by the local rate of distractor occurrence. Previous studies with feature-singleton distractors have interpreted this “distractor-location effect” as evidence that the more likely location receives a stronger spatial suppression, which, under certain conditions (Zhang et al., 2019), can reveal also a “target-location effect.” In the next experiment we addressed whether the “target-location effect” emerges also with abrupt onset distractors, but for the moment it is worth noting that with regard to the “distractor-location effect” the present study also confirmed that this effect takes time to vanish once the probabilities of distractor occurrences are equalized across locations, thus replicating what has been found with feature-singleton distractors (e.g., Duncan & Theeuwes, 2020; Valsecchi & Turatto, 2021).

## Experiment 2

To test whether different degrees of habituation to onsets are accompanied also by the “target-location effect,” we slightly modified the test phase of the previous experiment, while the training phase remained identical. During the test phase participants were asked to report the side, left versus right, of the gap present in the same onset annulus used as distractor during training. So, during the test phase the target became the previous onset distractor. We have recently shown that with feature-singleton distractors this manipulation allows to detect reliable target processing impairment at the previous high-probability distractor location (Turatto & Valsecchi, 2021). In addition, since during training the locations of the distractor were different from those of the target, by presenting in the test phase the target at the previous distractor locations we could directly investigate lingering effects of distractor filtering on target processing uncontaminated by any previous unbalanced spatial target probabilities, which, however, were equally distributed among locations.

### Methods

#### Participants

Participants were recruited with the same criteria and procedures as for Experiment 1. The data of 36 participants were collected to match the sample size of Experiment 1. None of the datasets had to be rejected because the minimum accuracy requirement of 85% of correct responses in either the training or test phase was reached. Participants were paid 3.75 GBP for their participation and the experiment lasted approximately 30 min.

#### Stimuli and procedure

**Fig. 3:**
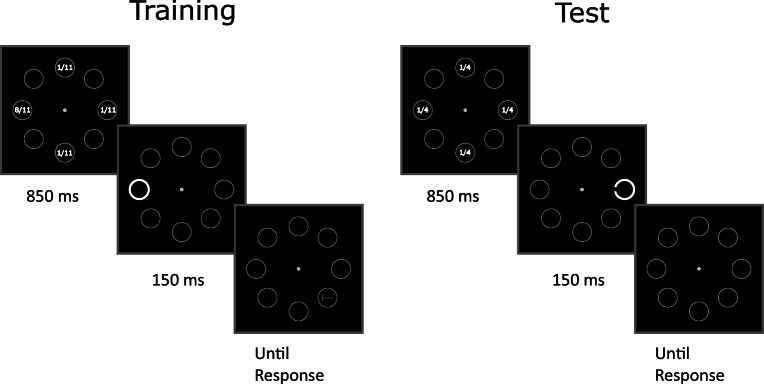
Stimuli and procedure of Experiment 2. During training participants reported the orientation of the target letter T (left vs. right) while ignoring the onset distractor appearing on 50% of the total trials. The locations of the target and distractor were completely segregated within and between trials. The target could appear only in the placeholders located along the oblique meridians, whereas the distractor could appear only in the placeholders located along the vertical and horizontal meridians, and it was 8 times more likely to appear in one of the four possible locations (the numbers inside the placeholders indicate the distractor rate, and were not shown during the experiment). In the example depicted, the higher distractor rate coincided with the left position along the horizontal axis. In the test phase, participants reported the orientation of the gap (left or right) in the abrupt onset annulus, which appeared at the same locations of the previous distractors during training (along the vertical or horizontal meridians), but now with equal probability at all locations

The stimuli and procedure in the training phase were as in Experiment 1 (Fig. 1). By contrast, in the test phase the peripheral-onset annulus, which appeared on every trial, became the target stimulus (Fig. 3). The annulus had a gap either on the left or on the right side (15 pixels wide) and participants were required to press as quickly and accurately as possible either the “G” or “H” key on the keyboard in order to indicate the side (left vs. right) of the gap. When participants responded incorrectly or failed to respond within 2,500 ms, they received a warning message. The onset annulus target appeared with the same probability in the four positions previously occupied by the same stimulus when it was a distractor in the training phase.

Participants underwent a practice phase of 28 trials, followed by a training phase of 352 trials and a test phase of 176 trials, and they were allowed to rest after performing 176 training trials, and between the training and test phases. The instructions for the test phase were given before the phase began and repeated after the first 16 test trials. In the training phase, the onset distractor appeared on 50% of the trials and was eight times more likely to appear at one location (balanced across participants) compared to each of the remaining three locations. This yielded 176 trials without distractors, 128 trials with the distractor at the high-probability location and 48 trials with the distractor at a low-probability location. In the test phase the target onset appeared with equal probability at all four locations (44 trials at each location).

### Results and discussion

#### Training phase

**Fig. 4:**
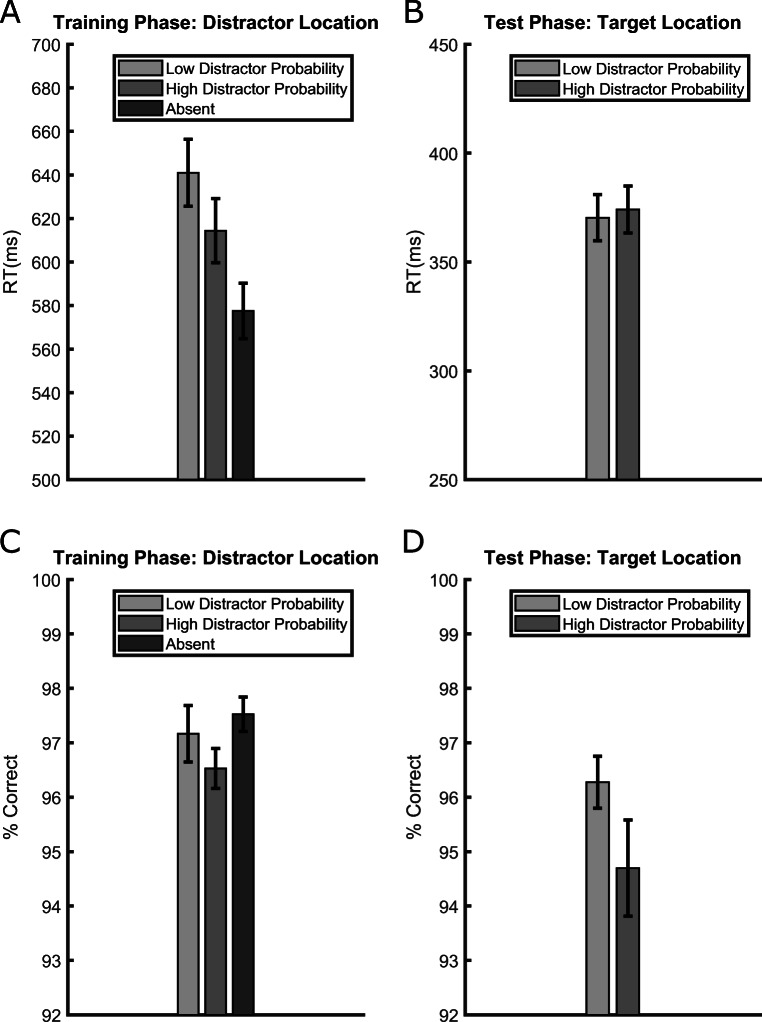
Average reaction times (RTs) (**upper panels**) and accuracies (**lower panels**) in the training (**left panels**) and test (**right panels**) phases of Experiment 2. Data from the training phase are presented separately for trials in which the distractor appeared at a low-probability location, at the high-probability location or was absent. Data for the test phase are separated based on the location where the target (the abrupt onset annulus with the gap) appeared, which could be either one of the low-probability distractor locations or the high-probability distractor location of the training phase. Error bars are standard errors of the mean (SEMs)

RT data from correct trials were pre-processed with the same outlier-removal procedure used in Experiment 1. In the test phase, we rejected RTs faster than 150 ms because in the gap-discrimination task RTs were on average much shorter relative to the letter orientation task. Overall, we rejected 1.47% of the trials in the training phase and 2% of the trials in the test phase. The results from the training phase are presented in Fig. 4a. Consistent with the fact that the training phase was identical to that of Experiment 1, we fully replicated the same pattern of results. The distractor presence slowed down participants relative to its absence, both when the distractor appeared at a low-probability location, *t*(35) = 8.022, *p* < .001, *d*_*z*_ = 1.337, RT difference = 63 ms, and when it appeared at the high-probability location, *t*(35) = 6.531, *p* < .001, *d*_*z*_ = 1.088, RT difference = 37 ms. Crucially, RTs were longer when the distractor appeared a low-probability location compared to the high-probability location, *t*(35) = 6.541, *p* < .001, *d*_*z*_ = 1.09, RT difference = 27 ms, thus replicating the fact that distractor rejection was more efficient where the abrupt onset was more likely to appear.

Accuracy data from the training phase are presented in Fig. 4c. The results did not mirror those of RTs, as was the case in Experiment 1. In Experiment 2 accuracy was lower at the high-probability distractor location. The comparison between the high-probability location and distractor-absent trials was significant, *t*(35) = 2.765, *p* =.009, *d*_*z*_ = 0.461, accuracy difference = 0.99%, whereas the comparison between the low-probability location and distractor-absent trials, *t*(35) = 0.664, *p* = .511, *d*_*z*_ = 0.11, accuracy difference = 0.35%, and between the high- and low-probability location trials, *t*(35) = 1.432, *p* = .194, *d*_*z*_ = 0.161, accuracy difference = 0.63%, were not significant.

#### Test phase

Because in the test phase the target appeared at the previous distractor locations during the training phase, in Experiment 2 we had the opportunity to measure the “target-location effect.” We did not observe a significant difference in RTs based on whether the target appeared at the high- or low-probability distractor location during the previous training, *t*(35) = 1.083, *p* = .286, *d*_*z*_ = 0.18, RT difference = 4 ms (see Fig. 4b). To corroborate this finding, we also computed the corresponding Bayes factor, which indicated that the results were more than three times more likely to be originated in the absence of an effect *BF*_01_ = 3.252.

Although accuracy data (Fig. 4d) indicated that participants were 1.57% more accurate when responding to targets at the formerly low-probability distractor location, this comparison was not statistically significant, *t*(35) = 1.879, *p* = .068, *d*_*z*_ = 0.313 and neither of the two hypotheses was supported by the Bayes factor analysis *BF*_01_ = 1.1519.

#### Combined analysis of the training phase of Experiments 1 and 2

Given that in the training phase the experimental procedure and design were identical in both experiments, we decided to combine the two datasets to investigate the temporal development of the onset distractor filtering as a function of its rate of occurrence at a given location. To this aim, we pooled the data of all 72 participants, and divided the training phase into four bins of 88 trials each, which included 44 distractor-absent trials, 32 trials with the distractor at the high-probability locations and 12 trials with the distractor at a low-probability location. Given the small number of trials in the low-probability location for each participant and bin, we did not apply the adaptive outlier removal algorithm and instead opted to reject only RTs shorter than 200 ms, and we did not analyze the accuracy data.

**Fig. 5:**
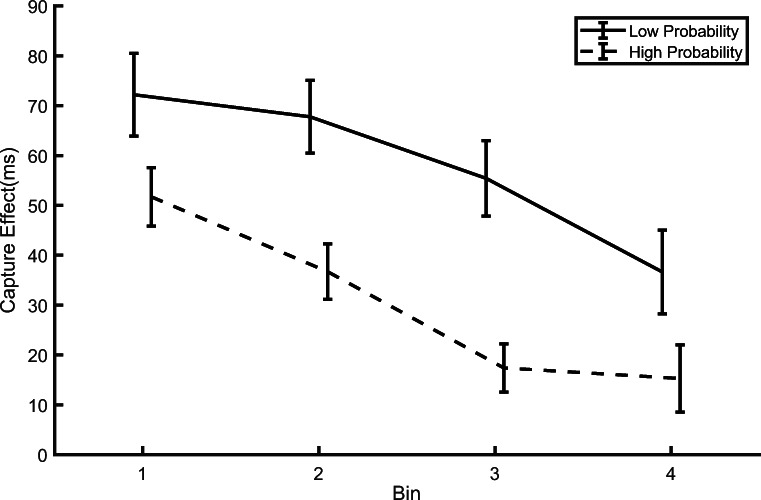
Temporal evolution of the capture effect (difference between reaction times (RTs) in distractor-present and distractor-absent trials) in the training phase of Experiments 1 and 2 combined. Each bin entails 88 trials. Error bars represent standard errors of the mean (SEMs)

Based on the binned data, we computed the capture effect,^1^ defined as the difference between RTs in distractor-present and distractor-absent trials, for each bin and for the high- and low-probability locations (Fig. 5). The results clearly indicated that irrespective of the spatial rate of distractor occurrence the amount of capture significantly decreased as training progressed, a typical sign of habituation. This was confirmed by a within-participants repeated-measures ANOVA, with Bin (1–4) and Location (High vs. Low distractor probability) as factors. This yielded a significant main effect of Bin, *F*(3, 213) = 10.879, *p* < .001, *ηp*^2^ = .132, with capture decreasing between Bins 1 and 4, RT difference = 36 ms, and Location, *F*(1, 71) = 59.994, *p* < .001, *ηp*^2^ = .458, RT difference = 28 ms, but no significant interaction, *F*(3, 213) = 1.562, *p* = .199, *ηp*^2^ = .021. We also performed a Bayes factor analysis using the JASP Version 0.16 software, which indicated that the data provided evidence against including the interaction to model the data (*BF*_*01*_=15.9). The decreasing capture observed in both conditions depicted in Fig. 5 is compatible with the habituation account, according to which the response elicited by an irrelevant stimulus is progressively attenuated, with more pronounced habituation the higher the rate of stimulation (Thompson, 2009). This decreasing RT pattern is also in line with our previous findings showing habituation to an onset distractor appearing, across trials, equally often at all locations (Turatto et al., 2018a, b, 2019; Turatto & Pascucci, 2016). In addition, the degree of habituation as a function of the distractor spatial rate of occurrence emerged very quickly, as RTs were shorter for distractors at the high-probability location relative to the low-probability location already in the first bin, *t*(71) = 2.833, *p* = .006, *d*_*z*_ = 0.333, RT difference = 20 ms.^2^ Lastly, it is worth noting that here we observed habituation of capture also when attention was not fully focused at the target location before the abrupt onset occurrence, as instead observed in our previous studies (e.g., Turatto & Pascucci, 2016).

## Experiment 3

**Fig. 6:**
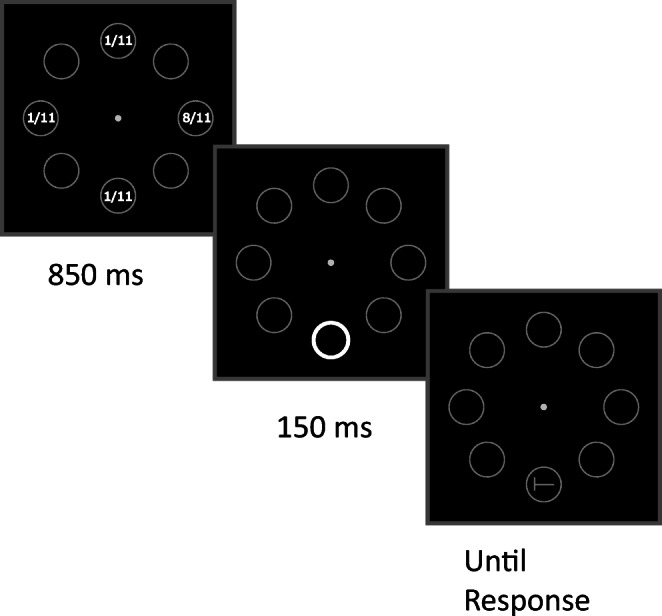
Stimuli and procedure of Experiment 3. During training participants reported the orientation of the target letter T (left vs. right) while ignoring the onset appearing on 50% of the total trials. The target and onset locations, which were placed along the vertical and horizontal meridians, overlapped in 25% of the trials. The onset was still eight times more likely to appear in one of the four possible locations (the numbers inside the placeholders indicate the distractor rate, and were not shown during the experiment), whereas the target was equally likely at all locations. In the example depicted, the higher distractor rate coincided with the right position along the horizontal axis

A reduced capture at the location with the higher rate of onset occurrence, and the fact that capture decreased with practice, are two findings consistent with the habituation account. However, because in the training phase of Experiments 1 and 2 the possible target and distractor locations were completely segregated, the capture reduction could potentially indicate that participants became progressively more efficient in attending the target locations. To rule out this alternative account, we conducted a third experiment where on each trial the target and the irrelevant onset could randomly and independently appear in one of the same four possible locations, thus preventing any segregation of the target and distractor locations (see Fig. 6). However, whereas the target occurred equally likely at all locations, the irrelevant onset was still eight times more likely at one of the four locations. This entails that the target and the irrelevant onset co-occurred at the same location on 25% of the trials. If the previous capture reduction reflected an improved selection of the target location, then under these conditions no evidence of capture decrement should emerge; by contrast, if the decrement was an instance of habituation, we expected to replicate the same finding. In fact, one could also predict a larger capture compared to previous Experiments 1 and 2, when the target and the distractor never shared the same location, as it might be more complicated to ignore a distractor that shares a spatial feature, like its location, with the target.

Furthermore, because of this spatial overlap, we had also the possibility to again test the “target-location effect,” if any, directly during the training phase, as in previous studies with feature-singleton distractors (e.g., Wang & Theeuwes, 2018).

### Methods

#### Participants

Participants were recruited with the same criteria and procedures used in Experiments 1 and 2. The data of 36 participants were collected to match the sample sizes of Experiments 1 and 2. None of the datasets had to be rejected for not reaching the minimum accuracy requirement of 85% of correct responses in either the training or test phase. Participants were paid 3.75 GBP for their participation, and the experiment lasted approximately 25 min.

#### Stimuli and procedure

The stimuli, procedure and experimental design were equivalent to those of the training phase of Experiments 1 and 2, with the only difference being that the target, as well as the onset distractor, were presented only in the locations along the vertical or horizontal meridians (see Fig. 6). In other words, the experimental procedure is equivalent to that of Experiment 1 or 2 with the exception of a clockwise or counterclockwise displacement of the target locations by one placeholder. The target was presented on each trial and with equal probability at all four locations, whereas the irrelevant onset appeared in 50% of all trials, but still 8 times more often at one of the four locations. On 25% of the trials where the onset was presented, it was followed by the target at the same location. In this respect, the paradigm resembled a classic Posner spatial-cueing task, where the onset acted like an exogenous uninformative cue.

The experimental design consisted of a practice phase of 28 trials, in which no onset was presented, followed by a training phase of 352 trials. Participants were allowed to rest after 176 training trials. The onset appeared at the high-probability location in 128 trials, and at a low-probability location in 48 trials. It overlapped with the target in 44 trials (25% of 176 trials), 32 at the high-probability location and 12 at a low-probability location. The target appeared equally likely at each location (88 trials), irrespective of whether the onset was absent or present.

### Results and discussion

Pre-processing of RT data involved the removal of the incorrect responses and the implementation of an outlier-removal procedure based on Median Absolute Deviation (Leys et al., 2013) for each participant and cell of the design, with a threshold of 5 MADs. We further removed RTs shorter than 200 ms from the analyses. Overall, we rejected 1.21% of the total trials.

#### Distractor-location effect

**Fig. 7:**
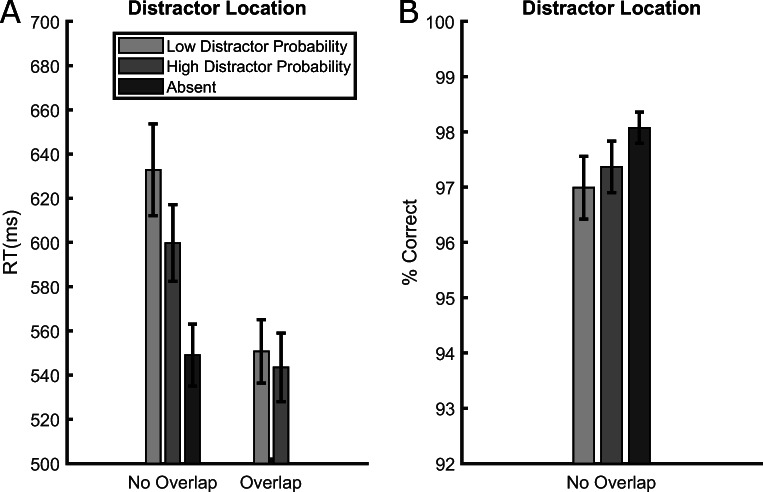
Average reaction times (RTs) (**A**) and accuracies (**B**) in Experiment 3. The data are presented separately for trials in which the distractor appeared at a low-probability location, at the high-probability location or was absent. RT data (**A**) are further divided based on whether the target and onset locations overlapped within a trial. Accuracy data (**B**) refer only to trials where the target and distractor positions did not overlap; accuracies in overlap trials were not computed given the small number of trials with overlap at the low-probability distractor location. Error bars represent standard errors of the mean (SEMs)

RT data from Experiment 3 are presented in Fig. 7a. In the no-overlap trials the results were equivalent to those of Experiments 1 and 2 during the training phase. Participants were slower in distractor-present trials relative to distractor-absent trials, when the distractor appeared both at a low-probability location, *t*(35) = 10, *p* < .001, *d*_*z*_ = 1.667, RT difference = 84 ms, and at the high-probability location, *t*(35) = 9.189, *p* < .001, *d*_*z*_ = 1.531, RT difference = 51 ms; in addition, the degree of capture was smaller when the distractor appeared at the high-probability location than at a low-probability location, *t*(35) = 5.979, *p* < .001, *d*_*z*_ = 0.996, RT difference = 33 ms.

By contrast, RTs in overlap trials did not differ from distractor-absent trials^3^, and this both at the low-probability location, *t*(35) = 0.306, *p* = .761 *d*_*z*_ = 0.051, RT difference = 2 ms, and at the high-probability location, *t*(35) = 1.363, *p* = .181 *d*_*z*_ = 0.22, RT difference = 5 ms. Also, in the overlap trials RTs did not differ significantly between target at the low- and vs. high-probability location, *t*(35) = 1.247, *p* = .22 *d*_*z*_ = 0.208, RT difference = 7 ms. Although we may have expected to find a target processing benefit when the onset (cue) appeared at the target location compared to when no onset was presented, it is worth nothing that in exogenous spatial-cueing tasks it is not uncommon to find an RT difference between congruent and incongruent trials, but no difference between congruent and cue-absent trials (e.g., Folk & Remington, 1998; Remington et al., 1992).

Accuracy data from trials where no overlap between onset and target occurred are presented in Fig. 7b. Similar to the findings of Experiment 1, the results show an opposite pattern to the one that emerged for RTs, excluding a possible speed-accuracy trade-off. However, possibly due to the very high overall accuracy, none of the comparisons turned out to be significant: low-probability location versus distractor-absent, *t*(35) = 1.976, *p* = .056, *d*_*z*_ = 0.329, accuracy difference = 1.08%; high-probability location vs. distractor-absent, *t*(35) = 1.699, *p* = .098, *d*_*z*_ = 0.283, accuracy difference = 0.7%; low- vs. high-probability location, *t*(35) = 0.514, *p* = .61, *d*_*z*_ = 0.085, accuracy difference = 0.37%. We did not perform an analysis of accuracy on trials where the onset and the target overlapped given the small number of this type of trials at the low-probability distractor location.

#### Target-location effect

**Fig. 8:**
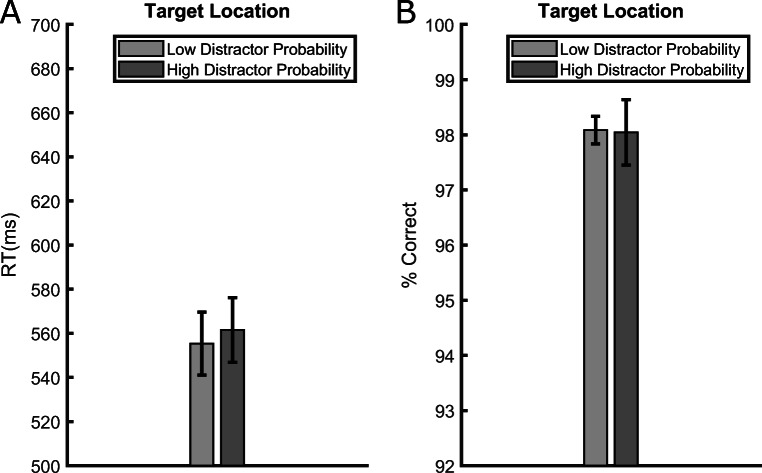
Average reaction times (RTs) (**A**) and accuracies (**B**) in trials without onset in Experiment 3. The data are presented separately for trials in which the target appeared at a low- or high-distractor probability location. Error bars represent standard errors of the mean (SEMs)

Because on some trials the target and the distractor shared the same location, we had the opportunity to evaluate the “target-location effect” directly during training, whereas in Experiment 2 this effect could be evaluated only in the test phase by presenting the target in the previous distractor locations. In other words, here we had the opportunity to ascertain whether the local rate of distractor occurrence affected the processing of the target at the same location on distractor-absent trials, as usually reported in previous studies with feature-singleton distractors (Ferrante et al., 2018; Goschy et al., 2014; Sauter et al., 2018; Wang & Theeuwes, 2018). Figure 8a shows that while the RT difference (6 ms) was in the expected direction, i.e. longer RTs for targets appearing at the high-probability distractor location compared to the low-probability distractor locations, the difference failed to reach significance, *t*(35) = 1.833, *p* = .075, *d*_*z*_ = 0.305, thus replicating what we observed in Experiment 2 during the test phase, although in this case the Bayes factor analysis yielded an undecided result *BF*_01_ = 1.239. The corresponding accuracy data (Fig. 8b) also did not show a significant effect of the local distractor probability, *t*(35) = 0.078, *p* = .938, *d*_*z*_ = 0.013, accuracy difference = 0.042%, *BF*_01_ = 5.57. In other words, we again failed to find robust evidence supporting the “target-location effect,” so the issue of whether this effect is detectable with abrupt onset distractors remains at present undecided.

Although the Bayes factor analysis indicated that we could not reach a decisive answer with regard to the “target-location effect,” still one could speculate that one reason why in Experiment 2 we may have failed to replicate this finding could be that the context of search was quite different between the training and test phase. Indeed, during training participants searched for a target T while being exposed to an onset annulus distractor, whereas in the test phase the context of search was defined by the same onset annulus becoming the target. However, the possibility that such task-context change might have prevented the “target-location effect” to emerge seems less likely in light of the results of the present experiment, where we measured the “target-location effect” during training, namely in the same task context, similarly to what is usually done in feature-singleton distractors studies. Alternatively, as argued in the *General discussion*, the lack of a “target-location effect” might be accounted for by the fact that in our paradigm onset rejection was not achieved via spatial suppression exerted at the saliency map level; rather, since the distractor was always a white bright transient element and did not change across trials, suppression may have been exerted at dimension-specific map level (Liesefeld & Müller, 2021; Zhang et al., 2019).

#### Learning to ignore the distractor

**Fig. 9:**
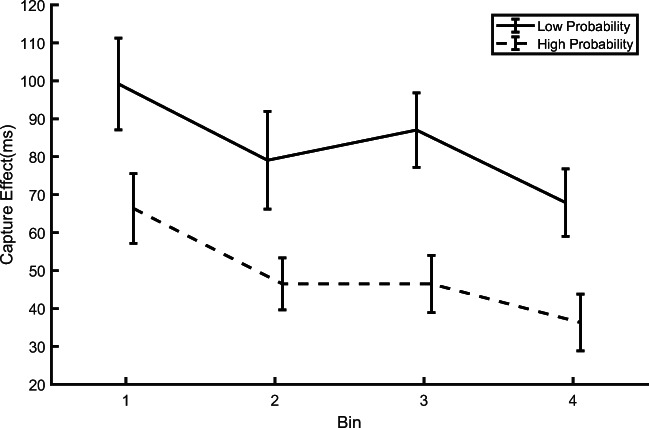
Temporal evolution of the capture effect (difference between reaction times (RTs) in distractor-present and distractor-absent trials) in Experiment 3. Each bin entails 88 trials. Error bars represent standard errors of the mean (SEMs)

If the reduction of capture across trials found in Experiments 1 and 2 was not an instance of habituation, but reflected instead the fact that participants progressively learned to better select the target locations with respect to the distractor locations, which was favored by their complete segregation, then their partial overlap (occurring on 25% of the trials) in the current experiment should have prevented any attenuation of capture. To test this possibility, similar to what we did when we analyzed the combined training data of Experiments 1 and 2 (Fig. 5), we performed an analysis of the evolution of the capture by splitting the data of Experiment 3 into four bins of 88 trials each^4^. As in the previous analyses of the “distractor-location effect,” we excluded the trials where target and onset overlapped at the same location. The pattern of results depicted in Fig. 9 appears similar to the training phase of Experiments 1 and 2, with a reduction of capture from Bin 1 to Bin 4, both for the high- and for the low-probability distractor locations. This was confirmed by a within-participants repeated-measures ANOVA, with Bin (1–4) and Location (High- vs. Low-distractor probability) as factors, which yielded a significant main effect of Bin, *F*(3, 105) = 5.121, *p* = .002, *ηp*^2^ = .127, with capture decreasing from Bin 1 to Bin 4, RT difference = 31 ms, and Location, *F*(1, 35) = 33.169, *p* < .001, *ηp*^2^ = .486, RT difference = 34 ms, but no significant interaction, *F*(3, 105) = 0.276, *p* = .842, *ηp*^2^ = .007. The Bayes factor analysis indicated that the data provided evidence against including the interaction to model the data (*BF*_*01*_ = 22.49).

In order to directly compare the results from the training phase of Experiments 1 and 2 with the results from Experiment 3, we also performed a three-way repeated-measure ANOVA with Bin (1–4) and Location (High- vs. Low-distractor probability) as within-participants factors, and Experiment (1+2 vs. 3) as a between-participants factor. This confirmed that the effects of Bin and Location did not differ in the two experimental conditions, given that the three-way interaction was not significant, *F*(3, 318) = 0.243, *p* = .866, *ηp*^2^ = .002. The Bayes factor analysis indicated that the data provided evidence against including the three-way interaction to model the data (*BF*_*01*_ = 28.6). Crucially, however, the main effect of Experiment was significant, *F*(1,106) = 7.947, *p* = .006, *ηp*^2^ = .07, attesting that capture was overall larger in Experiment 3 as compared to Experiments 1+2, RT difference = 22 ms.

To summarize, the present experiment confirmed that: (a) in agreement with habituation, the efficiency of onset distractors rejection was proportional to the rate of onset occurrence at a given location; (b) regardless of the onset rate, capture diminished as exposure to the onset progressed, again in accordance with the habituation account; (c) we failed to find a reliable “target-location effect”. In addition, when the target and the onset distractor occurred at the same location distractor filtering was less efficient, as attested by the larger capture observed in Experiment 3 compared to Experiments 1 and 2, where their locations were completely segregated.

## General discussion

In previous studies we found that with practice the distracting effect of a peripheral visual onset appearing with equal probability at different locations was progressively attenuated, a result that we interpreted as evidence of habituation (Turatto et al., 2018a, b; Turatto & Pascucci, 2016). Here we have complemented these findings by showing that the amount of habituation to an onset distractor is modulated by its rate of occurrence at different locations, being larger where the distractor was more likely to appear; in addition, regardless of the rate of onset occurrence at a given location, capture was progressively attenuated with practice. These two findings are in agreement with the habituation of capture hypothesis, as habituation reflects a progressive response reduction that is proportional to the rate of stimulation (Thompson, 2009). By contrast, we did not find robust evidence suggesting that habituation of capture at a given location was achieved by means of spatial suppression at that location, as the efficiency of target processing was equivalent at the high- and low-probability distractor location.

The present paradigm is different from those typically used to study feature-singleton distractor rejection, often based on the additional-singleton paradigm where both the target and the distractor are static discontinuities that appear simultaneously embedded in the search display, and therefore a direct comparison of the results obtained might not be straightforward. Yet, it might be still worth also commenting on the present findings in relation to those of the previous studies on the SL of distractor location.

### The distractor-location effect

Among the different mechanisms that have been proposed for distractor filtering the spatial suppression of the distractor location seems to be a prominent one (Chelazzi et al., 2019; Geng et al., 2019; Liesefeld & Müller, 2019). This idea, emerged from studies using feature-singleton distractors occurring with different probabilities in different spatial regions (e.g., Goschy et al., 2014; Sauter et al., 2018), postulates that the amount of spatial suppression is controlled by a SL process that estimates the rate of distractor occurrence at a given location, and on the basis of this information a certain level of suppression is applied at the level of the saliency or dimension-specific maps (Ferrante et al., 2018; Wang & Theeuwes, 2018). By contrast, in the case of onsets others have suggested that the progressive attenuation of capture might be an instance of habituation (e.g., Turatto & Pascucci, 2016), although a habituation mechanism has been invoked to account for the reduced capture elicited by a color singleton distractor as well (De Tommaso & Turatto, 2019; Won & Geng, 2020).

These two accounts are usually presented as complementary but distinct mechanisms (e.g., Chelazzi et al., 2019; Geng et al., 2019; Luck et al., 2021), but are they really different? Framed in this way the question could be misleading, as SL refers to a process or mechanism to extract information from the history of stimulation, whereas habituation is a phenomenon consisting in the waning of the response elicited by a stimulus that is repeatedly presented. Rather, the appropriate theoretical question is whether SL and habituation mechanisms are different, and to try to answer this, the *stimulus-model comparator* theory proposed by Sokolov (1960, 1963) and the *gnostic-unit* theory that Wagner (1976, 1978) developed on the ground of the original idea of Jerzy Konorski (1967) are particularly relevant. The influential dual-process theory proposed by Groves and Thompson (1970), is less relevant because it originates from studies on the flexor withdrawal reflex to a repeated stimulation in the acute spinal cat, and explains habituation as the result of synaptic rather than cognitive mechanisms. 

In particular, Sokolov’s theory was elaborated to account for the habituation of the OR, which was mainly defined in terms of a constellation of electrophysiological, physiological and muscular responses, in addition to the orienting of attention, although at that time the spatial covert orienting of attention was not yet isolated and measured. The core of the theory relies on a comparison process between the current sensory input and a neural model of the past stimulation held in short-term memory (STM). When the result of the comparison is a mismatch, an OR toward the new stimulation is triggered; by contrast, the more the stimulus matches the neural model, the more the OR is inhibited or suppressed, and habituation arises. This is the usual way Sokolov’s theory is presented, which on the one hand makes immediately clear that habituation should not be confounded with a peripheral sensory adaptation process, but on the other hand it may convey the idea that habituation would result from a match between the sensory input and the model accrued from the past stimulation. In fact, Sokolov stressed one key but often overlooked aspect of his theory, namely that the comparison would occur between the current and the “expected” input, with the latter being “extrapolated” from the information accumulated in the neural model, namely on the basis of the history of stimulation. In other words, Sokolov’s model is basically governed by a “prediction error” rule, with the OR triggered by a discrepancy or error between the current input and the expected one, and conversely habituation develops as the prediction error is progressively reduced. The predictive nature of the neural model is clearly pointed out in several passages of Sokolov’s works: for example, in the 1960 paper it is reported that “*The nervous system thus elaborates a forecast of future stimuli as a result of repeated stimulation and compares these forecasts with the stimuli actually in operation*.” (Sokolov, 1960; Appendix, page 287), while in the 1963 paper Sokolov claimed that “*… the nervous model should not be conceived of simply as a passive stable engram, but as a mechanism which can extrapolate the patterning of future nervous impulses.*”(Sokolov, 1963; page 568). In addition, Sokolov was very explicit in postulating that the neural model “*… simultaneously represents the intensity, quality, and temporal characteristics of signals …*” (Sokolov, 1963; page 562), and that habituation thus relies on expectations or predictions based on the statistics of the past events. In particular, since Sokolov was mainly concerned with sequential patterns of stimuli presented in isolation, like the repetition of lights or tones, the statistics were mainly defined by the stimulus temporal frequency. However, there is no doubt that the model is governed by a SL process (and by a suppression mechanism), similar to that recently advocated to control the degree of suppression for a feature-singleton distractor that appears with different rates of occurrence at different locations (Ferrante et al., 2018; Wang & Theeuwes, 2018).

In brief, in Sokolov’s model the statistics that is learned is extracted from the sequence of stimulations, like for instance the stimulus temporal frequency or its contingency with other stimuli, which allows the neural system to develop expectations about future events. So, how could the Sokolov model, for example, account for the fact that the capture response is more attenuated for a distractor that appears more often at a given location? It should be noted that as compared to habituation studies in animals where the habituating stimulus is presented in isolation, in a typical attentional capture paradigm, and especially in the one used to study habituation of onset capture like that proposed here, the distractor is not the only salient event that is presented, but rather it appears with several other prominent and discrete visual events, like for example the fixation point, possible placeholders or non-target stimuli, and the target, which appear also in the absence of the distractor (i.e., the distractor-absent trials). Although the distractor rate is directly proportional to the distractor temporal frequency, it is very likely that in this kind of paradigm the main factor determining the distractor expectation is not its temporal frequency (Geer, 1966), but its contingency with the display, which may vary at different locations. Hence, because the occurrence of the distractor at the high-probability location is largely expected, it will generate a small prediction error or discrepancy with expectation, and the corresponding attentional response will be largely inhibited. By contrast, the distractor appearance at the low-probability location will be much less expected, thus leading to a larger prediction error, and a weaker capture response suppression and habituation.

Hence, a Sokolovian model that generates a distractor expectation on the basis of the learned statistics at specific or general locations can explain in a straightforward way both the fact that the amount of capture is larger the lower the overall distractor probability in the display (e.g., Turatto & Pascucci, 2016), and the present findings showing an overall reduced capture where the distractor was more likely to appear. In addition, it can also explain why the amount of capture shows a progressive reduction across trials, a typical sign of habituation. So, if the reduced capture observed at the more frequent onset location were not an instance of habituation, but were instead controlled by a different SL mechanism, then those who advocate this view should specify what kind of different predictions, if any, the latter makes with respect to the former. At present we see no clear ways to distinguish the two views, but since habituation mechanisms have been proposed more than half a century ago, and habituation has been documented in virtually all animal species and with a broad range of behavioral and neural responses, we prefer to interpret the present findings with onset distractors as an instance of habituation (also see Dukewich, 2009, for an explanation of the IOR in terms of habituation of the orienting of attention).

### The target-location effect

Previous studies with feature-singleton distractors have often shown that the stronger distractor filtering observed at the high-probability distractor location was accompanied by a larger impairment in target processing at the same location, as compared to where the distractor was less likely to occur (e.g., Ferrante et al., 2018; but see Zhang et al., 2019). This finding, known as “target-location effect,” would support the idea that space-based filtering can be achieved by applying suppressive signals at the feature-singleton location in the saliency or dimensions-specific map. The lingering suppressive effects then impair the subsequent processing of targets appearing at the distractor location (e.g., Wang & Theeuwes, 2018).

So, why is that with onsets we did not find robust evidence of the target-location effect? In Experiment 2, the target occurred at the previous distractor location only in the test phase, when the distractor was no longer presented, a condition that may have favored the extinction of any previous suppressive effect. This seems however unlikely, given that the test phase of our Experiment 2 was 176 trials long, which is comparable to the 144 trials of the test phase in the Di Caro and Della Libera (2021) study where a lingering “target-location effect” was observed. Furthermore, the test phase of Experiment 2 was shorter than the test phase in our previous study (216 trials), where a lingering suppression effect was also documented with feature-singleton distractors (Turatto & Valsecchi, 2021). Crucially, the extinction account is definitely ruled out by the lack of a reliable “target-location effect” in Experiment 3, where the target and the distractor appeared in the same four locations during training. Specifically, the two events appeared on the same location on congruent trials (25%), but they also occupied the same location across trials, similarly to what happens with the additional-singleton paradigm (e.g., Wang & Theeuwes, 2018), thus allowing a direct test of the effect during training. Yet, despite these favorable conditions no reliable evidence of “target-location effect” was found.

Perhaps a more viable explanation is provided by the results of Zhang et al.’s (2019) study, in which the authors have shown that when the distractor-defining feature was kept constant, like for example its color, the “target-location effect” disappeared. The authors have then proposed that under these conditions, participants may learn to suppress the distractor location at the dimension-specific maps level rather than at the saliency map level. The latter would then receive the already decreased activity at the distractor location from hierarchically lower maps, without implementing any suppressive signal that may affect the target selection at the distractor location (also see, Liesefeld & Müller, 2021). However, in our experiments target and distractor were both onset white stimuli, and consequently their respective saliency should have been represented within the same dimension-specific map (e.g., luminance), where the distractor spatial suppression should have resulted in any case in the “target-location effect.” At this point, the only way to explain the lack of such an effect is to make the post-hoc assumption that despite target and distractor were both white bright onset stimuli (especially in Experiment 2 where they were both white rings), they were in fact represented in different dimension-specific maps. Hence, at present the reason why we did not find evidence of the “target-location effect” with onset distractors is not entirely clear, especially because a null result (though replicated in two experiments) is often not very informative.

Our finding might, however, suggest that onsets may have a special status in the brain, and that although they can be ignored with practice, perhaps this is not primarily achieved by means of a spatial suppression exerted at the onset location in the saliency maps, but this is a speculation based on a result that we acknowledge needs to be replicated in further studies. The possibility that onsets may be different from feature-singletons is also suggested by the fact that the attenuation of capture elicited by an abrupt onset has been shown to be context specific, with a recovery of the habituated capture when the context is changed (Turatto et al., 2019; also see, Turatto et al., 2018a), in agreement with the fact that habituation can be context specific (Dissegna et al., 2021). By contrast, feature-singleton distractor rejection based on SL seems to generalize across different contexts (Britton & Anderson, 2020).

### Onset capture can be progressively attenuated

Here and in our previous studies (Bonetti & Turatto, 2019; Pascucci & Turatto, 2015; Turatto et al., 2018a, b, 2019; Turatto & Pascucci, 2016), we invariably found that the sudden occurrence of an irrelevant peripheral onset caused an unwanted capture of covert and overt attention that was progressively reduced. By contrast, in a recent study Ruthruff et al. (2019) found no evidence of capture attenuation as exposure to the irrelevant onset continued. There are several methodological differences between the paradigm adopted here and in our previous studies, and that used by Ruthruff and colleagues, which may potentially account for the different findings, but what seems a feasible explanation is that in the Ruthruff et al. (2019) study participants were submitted to a large number of onsets already during the practice phase (64 trials with ten onsets per trial), and habituation may have reached the asymptotic level during this phase. The method of presenting on each trial a flurry of onsets before the target was previously used by Pascucci and Turatto, who, however, reported a significant decrement of the peripheral onset interference on the central task as a function of the length of the onsets series, with a robust detrimental effect with one or two onsets and a complete habituation with four or five onsets (Pascucci & Turatto, 2015).

In sum, in three experiments we have shown that the distraction (interference in target discrimination) caused by a peripheral visual transient or onset can be attenuated as exposure to the irrelevant salient stimulation progresses, and that the distraction attenuation is governed by the specific spatial rate of onset occurrence. We interpreted our findings as evidence that onset capture is subject to spatially selective habituation, likely controlled by a Sokolovian mechanism based on expectation, which is determined, in our paradigm, by the statistics of the distractor occurrence at a given location.

## Footnotes

1 The RT Values in distractor-absent trials were on average 581.7, 582.2, 583.1, and 595.4 ms in blocks 1–4. The minimal trend towards increasing RTs across blocks was not significant (F(3,213)= 1.727 , *p* = .162, *ηp*^2^ = .023). This indicates that the decrease in capture was due to a reduction of the distraction effect per se, and does not mirror a proportional reduction of RT costs in the context of accelerating RTs as the experiment progressed.

2 Limited to the data from Experiment 1, we also performed a binned analysis of the results from the test phase. We split the data in two 96 trial bins and performed an ANOVA with Bin (1–2) and Location (High vs. Low distractor probability) as factors. This did not yield a significant Bin × Location interaction, *F*(1,35) *=* 2.226 *p* = .144, *ηp*^2^ = .059, and the difference between High and Low probability locations increased from 7 to 27 ms between Bin 1 and Bin 2, indicating that learning was rather resistant to extinction throughout the test phase.

3 Notice that we did not apply the MAD outlier rejection procedure to trials with onset-target overlap, given that the number of trials per cell is too small, but we did apply it to trials with overlap, so as to be consistent with the analysis of results in Experiments 1 and 2. We checked in a separate analysis that the same pattern of results (no significant difference between distractor-absent and overlap trials) holds also when no MAD rejection analysis is applied to the distractor-absent trials.

4 RTs in distractor-absent trials varied minimally across blocks (mean RTs were 558.4, 558.9 , 557.7, and 552.1 ms in blocks 1–4). The effect was not significant, *F*(3, 105) = 0.43, *p* = . 731, *ηp*^2^ = .012.
